# Diagnosis and treatment protocol for COVID‐19 patients (Trial Version 9)

**DOI:** 10.1002/hcs2.1

**Published:** 2022-07-12

**Authors:** Released by National Health Commission & National Administration of Traditional Chinese Medicine on March 15, 2022

**Keywords:** COVID-19, diagnosis, treatment, nursing, discharge criteria, clinical guideline

## Abstract

To further improve the diagnosis and treatment of COVID‐19, China National Health Commission and the National Administration of Traditional Chinese Medicine convened a group of experts to revise the relevant content of the Diagnosis and Treatment Protocol for COVID‐19 Patients (Trial Version 8) and developed the Diagnosis and Treatment Protocol for COVID‐19 Patients (Trial Version 9), summarizing the etiological characteristics, epidemiological characteristics, pathological changes, clinical features, diagnosis, clinical classification, population with high risk of severe/critical illnesses, early warning predictors for severe/critical illnesses, differential diagnosis, case identification and reporting, treatment, nursing, discharge criteria and precautions after discharge, patient transfer, control of nosocomial infection in medical institutions, and disease prevention.

**Abbreviations**
ACE‐2: angiotensin converting enzyme 2
COVID‐19: coronavirus disease 2019
CRP: C‐reactive protein
CRRT: continuous renal replacement therapy
ECMO: extracorporeal membrane oxygenation
ESR: erythrocyte sedimentation rate
FiO_2_
: fraction of inspired oxygen
HFNC: high‐flow nasal cannula/nasal high‐flow oxygen
IL‐6: Interleukin‐6
IVIG: intravenous immunoglobulin
NIV: non‐invasive ventilation
PaO_2_
: partial pressure of oxygen
PT‐PCR: real‐time reverse transcription‐polymerase chain reaction
RdRp: RNA‐dependent RNA polymerase
RR: respiratory rate
SpO_2_
: pulse oxygen saturation
TCM: traditional Chinese medicine
VA‐ECMO: venous‐arterial ECMO
VAV‐ECMO: veno‐arterial‐venous EMCO
VOC: variants of concern
VV‐ECMO: venous‐venous ECMO
WHO: World Health Organization

## INTRODUCTION

To further improve the diagnosis and treatment of COVID‐19, the China National Health Commission and the National Administration of Traditional Chinese Medicine convened a group of experts to revise the relevant content of the *Diagnosis and Treatment Protocol for COVID‐19 Patients (Trial Version 8)* and developed the *Diagnosis and Treatment Protocol for COVID‐19 Patients (Trial Version 9)*. The Protocol was released on March 15, 2022.

## 1 ETIOLOGICAL CHARACTERISTICS

The 2019‐nCoV (also as SARS‐CoV‐2) belongs to the beta genus of coronaviruses. It has an envelope, round or oval particles, and a diameter of 60–140 nm. It has five essential genes respectively targeting RNA‐dependent RNA polymerase (RdRp) and four structural proteins of nucleoprotein (N), envelope protein (E), matrix protein (M), and spike protein (S). The N protein wraps the RNA genome to form a nucleocapsid, which is surrounded by an E that contains the M and the S proteins. The S protein enters the cell by binding to angiotensin‐converting enzyme 2 (ACE‐2). When isolated and cultured in vitro, the 2019‐nCoV can be found in human respiratory epithelial cells in about 96 h, while it takes about 4–6 days to isolate and culture in Vero E6 and Huh‐7 cell lines.

The 2019‐nCoV, like all other viruses, mutates, and certain mutations may affect its biological characteristics. For example, the change in the binding affinity of the spike protein and ACE‐2 may affect the virus's ability of cell invasion, replication, and transmission, as well as the period of recovery, antibodies produced after vaccination, and the neutralizing ability of antibody therapeutics. Therefore, such mutation has attracted wide attention. There are five "variants of concern" (VOC) defined by the World Health Organization (WHO), namely Alpha, Beta, Gamma, Delta, and Omicron. At present, the Omicron variant has quickly replaced the Delta variant to become the dominant variant. Currently, available evidence shows that the Omicron variant is more transmissible than the Delta variant, but with weakened pathogenicity. The Omicron variant does not impact the SARS‐CoV‐2 detection capability of real‐time reverse transcription‐polymerase chain reaction (RT‐PCR) assays diagnostic, but it may reduce the neutralizing effect of some monoclonal antibody drugs.

Coronavirus is sensitive to ultraviolet rays and heat. 56°C for 30 min alone, ether, 75% ethanol, chlorine‐containing disinfectant, peracetic acid, chloroform, and other lipid solvents can effectively inactivate the virus, while chlorhexidine cannot.

## 2 EPIDEMIOLOGICAL CHARACTERISTICS

### 2.1 Source of infection

The source of infection is mainly patients infected with the 2019‐nCoV as well as asymptomatic carriers. Patients are infectious during the incubation period and are highly infectious within 5 days after the onset of the disease.

### 2.2 Route of transmission

a. The main route of transmission of 2019‐nCov is respiratory droplet transmission and close contact transmission.
b. The virus may spread through aerosols in a relatively closed environment.
c. Contact with items contaminated by the virus can also cause infection.

### 2.3 Susceptible population

Everyone is susceptible to 2019‐nCoV. Infection or vaccination can acquire certain immunity.

## 3 PATHOLOGICAL CHANGES

The followings are pathological changes in major organs caused by the 2019‐nCoV, along with the testing results (excluding underlying diseases).

### 3.1 Lungs

In the early and mild lesions, serous fluid, fibrin exudation, and hyaline membrane formation can be seen in the alveolar cavity, and the inflammatory cells are mainly monocytes and lymphocytes. The alveolar septal capillaries were congested. With the progression and aggravation of the lesion, a large number of monocytes/macrophages and fibrin fill the alveolar space. Type II alveolar epithelial cells proliferate, and some shedding of cells occurs as well. Multinucleated giant cells are found, and red‐stained inclusion bodies are occasionally seen. It is easy to find pulmonary vasculitis, thrombosis (mixed thrombus, clear thrombus), and thromboembolism. Part of the epithelium of the bronchial mucosa in the lungs shed, and exudates and mucus are detected in the cavity. Exudate and mucus are seen in the small bronchi and bronchioles. Small bronchi and bronchioles are prone to mucus plugging. Focal hemorrhages are common in lung tissue, and hemorrhagic infarcts and bacterial and/or fungal infections can be seen. Partial alveolar hyperinflation, rupture of alveolar septa, or cyst formation are seen. Alveolar space exudate fleshy change and pulmonary fibrosis are found among the patients with long course of the disease.

Under the electron microscope, coronavirus particles are found in the bronchial mucosal epithelium and cytoplasm of type II alveolar epithelial cells. Immunohistochemical staining shows that 2019‐nCoV antigen immunostaining and nucleic acid detection are positive in some bronchial epithelial cells, alveolar epithelial cells, and macrophages.

### 3.2 Spleen, hilar lymph nodes, and bone marrow

The spleen atrophies. White pulp and the lymphocytes are reduced, and some of these cells are necrotic. Hyperemia is found in the red pulp and focal hemorrhage can occur. Macrophages in the spleen proliferate and phagocytosis is visible. Splenic anemic infarction can appear. Lymph nodes can have fewer lymphocytes and necrosis can be seen here. Immunohistochemical staining shows that CD4+ T and CD8+ T cells in the spleen and lymph nodes are reduced. Lymph node tissue can be positive for the 2019‐nCoV nucleic acid test, and immunostaining for the 2019‐nCoV antigen of macrophages is positive. Bone marrow hematopoietic cells may proliferate or decrease in number, and the proportion of red granules increases; hemophagocytosis is occasionally seen.

### 3.3 Heart and blood vessels

Some cardiomyocytes can show degeneration, necrosis, interstitial congestion, or edema, and monocyte, lymphocyte, and/or neutrophil infiltration. Occasionally, the 2019‐nCoV nucleic acid test is positive.

Endothelial cell shedding and intimal or full‐thickness inflammation can be observed in small blood vessels throughout the body. Mixed thrombosis, thromboembolism, and infarction in corresponding parts can be detected in blood vessels. Visible thrombosis can be seen in the microvessels of the main organs.

### 3.4 Liver and gallbladder

Hepatocyte degeneration and focal necrosis with neutrophil infiltration can be seen, as well as liver sinusoid congestion, lymphocyte, and monocyte cell infiltration in the portal area, and microthrombus formation. The gallbladder is fully expanded, with gallbladder mucosal epithelial shedding. The liver and gallbladder show positive nucleic acid tests for the 2019‐nCoV.

### 3.5 Kidneys

Glomerular capillary congestion and segmental fibrinoid necrosis are occasionally observed. Protein exudates are seen in Bowman's space. The proximal tubules have degeneration of the epithelium, with some necrosis and shedding, and the casts in the distal tubules are easily observed. The renal interstitium can be congested, and microthrombus is identifiable. Kidney tissue occasionally tests positive for the 2019‐nCoV nucleic acid.

### 3.6 Other organs

Brain tissue congestion and edema, some neuronal degeneration, ischemic changes and loss, and occasional phagocytic phenomenon and satellite phenomenon can be detected, along with visible infiltration of monocytes and lymphocytes in the perivascular space. The epithelium of the esophagus, stomach, and intestinal mucosa show degeneration, necrosis, and shedding to varying degrees, and the lamina propria and submucosal monocyte and lymphocyte infiltration are observed. Cortical cell degeneration, focal hemorrhage, and necrosis are evident in the adrenal glands. In the testes, the number of spermatogenic cells decreases in varying degrees, and Sertoli cells and Leydig cells show degeneration.

The 2019‐nCoV can be detected in the nasopharynx and gastrointestinal mucosa, testes, salivary glands, and other organs.

## 4 CLINICAL FEATURES

### 4.1 Clinical manifestations

The incubation period is 1–14 days, mostly 3–7 days. The main symptoms are fever, dry cough, and fatigue. Some patients may present with nasal congestion, runny nose, sore throat, decreased or lost sense of smell and/or taste, conjunctivitis, myalgia, and diarrhea. Severe patients often develop dyspnea and/or hypoxemia within 1 week after the onset. Critically ill cases can quickly progress to acute respiratory distress syndrome, septic shock, irreversible metabolic acidosis, coagulation dysfunction, and multiple organ failure. A very small number of patients may also have central nervous system involvement and macrovascular necrosis. It is worth noting that severe and critically ill patients may only have low to moderate fever, or even no fever during the course of the disease.

Patients with mild symptoms may present with low fever, mild fatigue, olfactory and gustatory disorders, and without pneumonia. Some patients may also have no obvious clinical symptoms after 2019‐nCoV infection.

Those who have been vaccinated and those infected with the Omicron variant are mainly asymptomatic and mild. Patients with clinical symptoms mainly manifest with upper respiratory tract infection symptoms, such as low to moderate fever, dry throat, sore throat, nasal congestion, and runny nose.

Most patients have a good prognosis, with a few patients being critically ill. Critically ill patients are usually elderly, those with chronic underlying diseases, women in late pregnancy and perinatal period, or obese people.

Symptoms in children are relatively mild. Some children and newborns may have atypical symptoms, such as vomiting, diarrhea, and other gastrointestinal symptoms, or only poor response and shortness of breath. A very small number of children may have multiple system inflammatory syndromes (MIS‐C) with Kawasaki disease‐like or atypical Kawasaki disease manifestation, toxic shock syndrome, or macrophage activation syndrome, mostly in the recovery period. The main manifestations are fever with rash, nonpurulent conjunctivitis, mucosal inflammation, hypotension or shock, coagulopathy, acute gastrointestinal symptoms, and so forth. Once this occurs, the condition can deteriorate sharply in a short period of time.

### 4.2 Laboratory diagnosis

#### 4.2.1 General testing

In the early stage of the disease, peripheral white blood cell counts, as well as pymphocyte count, are normal or decreased. Some patients may have increased liver enzymes, lactate dehydrogenase, muscle enzymes, myoglobin, troponin, and ferritin. C‐reactive protein (CRP) and erythrocyte sedimentation rate (ESR) is elevated and normal procalcitonin level in most patients. Severe and critically ill patients can show increased d‐dimer level, a progressive decrease in lymphocyte counts, and an increased level of inflammatory factors.

#### 4.2.2 Etiology and serological examination

##### 4.2.2.1 Pathogenic test

RT‐PCR testing of samples from nasopharyngeal and oropharyngeal swabs, lower respiratory tract samples, and feces. Nucleic acid testing can be affected by the course of the disease, specimen collection, testing processes, and testing reagents. To improve the diagnostic accuracy of testing, specimens should be collected in a standardized protocol and sent for inspection as soon as possible after collection.

##### 4.2.2.2 Serological examination

The 2019‐nCoV‐specific immunoglobulin M (IgM) and IgG antibodies are positive, and the positive rate is low within one week of onset.

Due to the positive judgment value of the reagent itself, or the presence of interfering substances in the body (rheumatoid factor, heterophilic antibody, complement, lysozyme, etc.), or the cause of the specimen (hemolysis of the specimen, bacterial contamination of the specimen, excessive storage time of the specimen, incomplete coagulation of the specimen), the antibody test may show false positives. Generally, serological testing is not used as a diagnostic basis alone, and comprehensive judgments must be made in conjunction with epidemiological history, clinical manifestations, and underlying diseases.

### 4.3 Chest imaging

In the early stage, multiple small patchy shadows and interstitial changes are seen, especially in the periphery of the lung. Then it develops into multiple ground glass shadows and infiltration shadows in both lungs. Severe cases can show lung consolidation, but pleural effusion is rare. Patients with MIS‐C and cardiac dysfunction can show enlarged heart shadows and pulmonary edema.

## 5 DIAGNOSIS

### 5.1 Diagnostic principles

Diagnosis should be made on a basis of a comprehensive analysis of epidemiological history, clinical manifestations, and laboratory tests. A positive nucleic acid test for the new coronavirus is the primary criterion for diagnosis. For those who have not been vaccinated against the 2019‐nCoV, the detection of 2019‐nCoV‐specific antibodies can be used as a reference for diagnosis. For those who have been vaccinated against or infected with the 2019‐nCoV, in principle, antibodies are not used as the basis for diagnosis.

### 5.2 Diagnostic criteria

#### 5.2.1 Suspected cases

Have any of the following epidemiological histories and meet any two clinical manifestations; if there is no clear epidemiological history, meet three of the clinical manifestations; or have any two of the clinical manifestations and the 2019‐nCoV‐specific IgM antibody is positive (not used for recently vaccinated persons).

##### 5.2.1.1 Epidemiological history

a. Travel or residence history in a community with case reports within 14 days before the onset;
b. A history of contact with patients with 2019‐nCoV infection within 14 days of the onset;
c. Contact with patients with fever or respiratory symptoms from communities with case reports within 14 days of the onset;
d. Clustering disease (two or more cases of fever and/or respiratory symptoms occurring in small areas, such as homes, offices, and schools) within 14 days.

##### 5.2.1.2 Clinical manifestations

a. Fever and/or respiratory symptoms and other abovementioned clinical manifestations related to COVID‐19;
b. Have the abovementioned imaging characteristics of COVID‐19;
c. The total number of white blood cells is low or normal at the early stage, and lymphocyte count is low or normal.

#### 5.2.2 Confirmed cases

Suspected cases with any one of the following etiological or serological evidence:

a. A positive real‐time RT‐PCR detection of the 2019‐nCoV nucleic acid;
b. The 2019‐nCoV‐specific IgM and IgG antibody tests are positive for the unvaccinated person.

## 6 CLINICAL CLASSIFICATION

### 6.1 Mild

The clinical symptoms are mild and there is no pneumonia manifestation in imaging.

### 6.2 Moderate

With the above clinical manifestations, imaging showed pneumonia.

### 6.3 Severe

Adults meet any one of the following:

a. Shortness of breath, respiratory rate (RR) ≥ 30 times/min;
b. In the resting state, the pulse oxygen saturation (SpO_2_) is ≤93% while breathing ambient air;
c. Arterial partial pressure of oxygen (PaO_2_)/the fraction of inspired oxygen (FiO_2_) ≤300 mmHg (1 mmHg = 0.133 kPa);
  In areas with high altitudes (more than 1000 m above sea level), PaO_2_/FiO_2_ should be adjusted according to the following formula: PaO_2_/FiO_2_ × (760/atmospheric pressure [mmHg]).
d. The clinical symptoms are progressively worse, and lung imaging shows that the lesion has progressed significantly >50% within 24–48 h.

Children meeting any of the following:

a. High fever lasting more than 3 days;
b. Shortness of breath (<2 months old, RR ≥ 60 beats/min; 2–12 months old, RR ≥ 50 beats/min; 1–5 years old, RR ≥ 40 beats/min; >5 years old, RR ≥ 30 times/min), the influence of fever and crying excluded;
c. In the resting state, the pulse oxygen saturation is ≤93% while breathing ambient air;
d. Respiratory distress (nostril flapping, three concave signs);
e. Drowsiness, convulsions;
f. Refusal to feed or feeding difficulties with signs of dehydration.

### 6.4 Critical

Meet any one of the following conditions:

a. Respiratory failure and mechanical ventilation;
b. Shock;
c. Intensive care unit (ICU) admission due to other organ failures.

## 7 POPULATION WITH A HIGH RISK OF SEVERE/CRITICAL ILLNESSES

a. Older than 60 years;
b. Have comorbidities, such as cardiocerebrovascular diseases (including hypertension), chronic lung diseases, diabetes, chronic liver or kidney disease, and tumors.
c. Immune function deficiency (acquired immunodeficiency syndrome patients, long‐term use of corticosteroids, or other immunosuppressive drugs that lead to immune suppression);
d. Obesity (body mass index ≥ 30);
e. Late pregnancy and perinatal women;
f. Heavy smokers.

## 8 EARLY WARNING PREDICTORS FOR SEVERE/CRITICAL ILLNESSES

### 8.1 Adults

The following indicators should be alert to the deterioration

a. Hypoxemia or progressive exacerbation of respiratory distress;
b. Deterioration of tissue oxygenation (e.g., mixed venous oxygen saturation) or progressive increase in lactate;
c. Progressive decrease of peripheral blood lymphocyte count or an increase in inflammatory markers, such as interleukin‐6 (IL‐6), CRP, and ferritin;
d. Significant increase of d‐dimer and coagulation dysfunction;
e. Chest imaging showed an obvious progression of pneumonia.

### 8.2 Children

a. Increased breathing rate;
b. Poor response and lethargy;
c. Progressive increase in lactate;
d. Significant increase of CRP, procalcitonin, ferritin, and other inflammatory markers;
e. Bilateral or multiple lung lobes infiltration, pleural effusion, or rapid progression of the disease in a short period of time in imaging;
f. Underlying diseases (congenital heart disease, bronchopulmonary dysplasia, respiratory malformations, abnormal hemoglobin, severe malnutrition, etc.), immunodeficiency (long‐term use of immunosuppressive agents), and newborns.

## 9 DIFFERENTIAL DIAGNOSIS

a. The mild manifestations of 2019‐nCoV pneumonia must be differentiated from upper respiratory tract infections caused by other viruses.
b. Pneumonia caused by 2019‐nCoV needs to be distinguished from other known viral types of pneumonia, including influenza virus, adenovirus, respiratory syncytial virus, and mycoplasma pneumonia infection. Especially for suspected cases, rapid antigen testing and multiple PCR nucleic acid detection should be used as much as possible for differential testing of common respiratory pathogens.
c. Differentiation from noninfectious diseases, such as vasculitis, dermatomyositis, and organizing pneumonia.
d. Children with rashes and mucosal damage should be differentiated from Kawasaki disease.
e. The close contact of COVID‐19 must immediately receive etiological testing, even with a positive result of a common respiratory pathogen.

## 10 CASE IDENTIFICATION AND REPORTING

After identifying a suspected case or a positive case that meets the case definition, healthcare facilities at all levels should immediately collect specimens for 2019‐nCoV nucleic acid testing or immediately transfer suspected cases to designated hospitals under the premise of ensuring the safety of transfer and single‐person and single‐room isolation treatment. For the one with positive PCR results, he/she should be isolated in the designated site or sent to the designated hospital for treatment, and be reported directly online in accordance with the regulation.

Suspected cases with negative results for two consecutive nucleic acid tests (at least 24 h apart) can be ruled out for infection.

## 11 TREATMENT

### 11.1 Isolation and management based on the severity of the condition

a Mild cases are centralized and isolated. The centralized isolation places cannot be used for inbound personnel, close contacts, and other groups at the same time. During the isolation period, patients receive symptomatic treatment and monitoring. If the condition worsens, it should be transferred to a designated hospital for treatment.
b. Moderate, severe, critical cases, and cases with high‐risk factors of critically ill should be treated in the designated hospitals. Severe and critical cases should be admitted to ICU as early as possible, and patients with high‐risk factors and severe tendencies also should be admitted to ICU.

### 11.2 General treatment

a. Bed rest, supportive treatment, ensure adequate energy and nutrition intake, and pay attention to the balance of water and electrolyte to maintain homeostasis.
b. Closely monitor vital signs, especially oxygen saturation in rest and after activities.
c. Monitor blood routine, urine routine, CRP, biochemical variables (liver enzymes, myocardial enzymes, kidney function, etc.), blood coagulation function, arterial blood gas analysis, chest imaging, and so on, according to the condition. If possible, cytokine testing is feasible.
d. Supply effective oxygen therapy, including nasal cannula, mask oxygen, and nasal high flow oxygen therapy (HFNC) according to patient condition.
e. Antibacterial treatment: Avoid inappropriate use of antibacterial drugs, especially the combined use of broad‐spectrum antibacterial drugs.

### 11.3 Antiviral treatment

#### 11.3.1 Nirmatrelvir/ritonavir tablet (Paxlovid)

Indicators are mild‐to‐moderate adult COVID‐19 patients within 5 days of disease onset and high‐risk factors of severe disease. Usage: 300 mg (two 150 mg tablets) of nirmatrelvir with one 100 mg tablet of ritonavir, given twice daily for 5 days. The drug instructions should be read carefully before use, and it should not be used in combination with drugs, such as meperidine and ranolazine, which are highly dependent on CYP3A for clearance and whose plasma concentration increases can lead to serious and/or life‐threatening adverse reactions.

#### 11.3.2 Monoclonal antibodies

Ambavirumab/Romisevirumab Injection. Used in combination for adults and adolescents (12–17 years old, weight ≥ 40 kg) with mild to moderate COVID‐19 and high‐risk factors of severe disease. Usage: two drugs are administered at a dose of 1000 mg, respectively. After being diluted with 100 ml of normal saline, the two drugs are administered by intravenous sequential infusion at a rate of not higher than 4 ml/min, and 100 ml of normal saline is used to flush the tube in between. Clinically, monitor patients during infusion,  at least 1 h after infusion.

#### 11.3.3 Intravenous immunoglobulin (IVIG)

IVIG therapy for COVID‐19 can be used among high‐risk rapidly deteriorating patients with high viral load in the early stage of the disease. The recommended dosage for the mild case is 100, 200 mg/kg for the moderate case, and 400 mg/kg for severe illness. The second infusion can be administered the next day, depending on the improvement of the patient's condition, with the total number not exceeding 5.

#### 11.3.4 Convalescent plasma

It can be used in patients with high‐risk factors, a high viral load, and rapid disease progression in the early stage of the disease. The dosage is 200–500 ml (4–5 ml/kg), and whether to administer should be determined according to the individual patient's situation and viral load.

### 11.4 Immunotherapy

#### 11.4.1 Glucocorticoid

For patients with progressive deterioration of oxygenation, rapid imaging progression, and excessive inflammatory responses, glucocorticoids can be used for a short duration (3–5 days, and no more than 10 days). The recommended dosage is 5 mg/day for dexamethasone and 40 mg/day for methylprednisolone. Larger doses and long‐term use of glucocorticoids should be avoided to reduce side effects.

#### 11.4.2 IL‐6 inhibitor: Tocilizumab

Eligible for patients with severe and critical patients with elevated IL‐6 levels. Specific usage: The first dose is 4–8 mg/kg, with the recommended dose of 400 mg, 0.9% saline to dilute to 100 ml, and the infusion time of more than 1 h. If the first dose is not effective, the same dose can be applied 12 h after the first dose. The maximum number of administrations is 2 and the maximum single dose does not exceed 800 mg. Allergic reactions must be monitored. Contraindicated in active infections, such as tuberculosis.

### 11.5 Anticoagulation

For the patients with moderate symptoms and high‐risk factors and rapid disease progression, severe and critically ill patients, therapeutic doses of low molecular weight heparin or unfractionated heparin should be used when there is no contraindication. Treatment should be provided in case of thromboembolism according to the guidelines.

### 11.6 Prone position

The patients with moderate symptoms, high‐risk factors, and rapid disease progression, severe and critically ill patients should be given standardized prone position therapy for longer than 12 h per day.

### 11.7 Psychological intervention

Patients often have tension and anxiety. Psychological counseling should be strengthened and supplemented with drug treatment if necessary.

### 11.8 Supportive treatment of severe and critical cases

#### 11.8.1 Treatment principle

Prevent and treat complications, treat underlying diseases, prevent secondary infections, and provide timely organ function support based on the above management.

#### 11.8.2 Respiratory support

##### 11.8.2.1 Nasal cannula or face mask oxygen therapy

Patients with PaO_2_/FiO_2_ lower than 300 mmHg should receive oxygen therapy immediately. Patients who receive oxygen supplements via a nasal cannula or face mask oxygen therapy must be closely monitored for 1–2 h. If respiratory distress and/or hypoxemia do not improve, nasal HFNC or noninvasive ventilation (NIV) should be used.

##### 11.8.2.2 Nasal HFNC or NIV

Patients with PaO_2_/FiO_2_ less than 200 mmHg should receive HFNC or NIV. We recommend that patients who receive HFNC or NIV should receive awake prone position ventilation for at least 12 h if there is no contraindication.

Some patients have a high risk of HFNC or NIV treatment failure. Any patients receiving HFNO and NIV should be monitored closely. If there is no improvement in hypoxemia or the frequency of breathing or patients have excessive tidal volume or excessive inspiratory effort, especially after the prone position treatment within 1‐2 h, invasive mechanical ventilation should be performed immediately.

##### 11.8.2.3 Invasive mechanical ventilation

If the PaO_2_/FiO_2_ is less than 150 mmHg, especially in patients with significantly enhanced inspiratory effort, endotracheal intubation and invasive mechanical ventilation should be considered. However, given the atypical clinical manifestations of hypoxemia in severe and critically ill patients, PaO_2_/FiO_2_ should be evaluated in real‐time in combination with clinical manifestations and organ functions for endotracheal intubation and invasive mechanical ventilation. It is worth noting that the delay of endotracheal intubation may cause greater harm.

Early and appropriate invasive mechanical ventilation is important for critically ill patients. Lung protective mechanical ventilation strategies must be implemented. Lung recruitment manipulation can be performed in patients with moderate to severe acute respiratory distress syndrome with a FiO_2_ higher than 50%. Lung recrutability should be considered when considering repeated pulmonary recruitment maneuvers. It should be noted that some patients with COVID‐19 have poor recrutiablity lung and high positive end‐expiratory pressure (PEEP) should be avoided.

##### 11.8.2.4 Airway management

We recommend using an active heating humidifier and loop heating guide wire if possible for airway humidification. Closed suction and tracheostomy suction, if necessary, are recommended. Airway clearance therapy, such as vibration expectoration, high‐frequency thoracic oscillation, and postural drainage is recommended. Passive and active activities should be performed as soon as possible to promote sputum drainage and pulmonary rehabilitation in the case of stable oxygenation and hemodynamics.

##### 11.8.2.5 Extracorporeal membrane oxygenation (ECMO)

ECMO indications: patients who receive the optimal mechanical ventilation conditions (FiO_2_ ≥ 80%, tidal volume of 6 ml/kg ideal body weight, PEEP ≥ 5 cmH_2_O, and no contraindications) and meet any one of the following criteria should be considered to implement ECMO as soon as possible:

a. PaO_2_/FiO_2_ < 50 mmHg over 3 h;
b. PaO_2_/FiO_2_ < 80 mmHg over 6 h;
c. Arterial blood pH<7.25 and PaCO_2_ > 60 mmHg for more than 6 h, and RR > 35 times/min;
d. RR > 35 times/min, arterial blood pH <7.2 and the plateau pressure >30cmH_2_O;

Critically ill patients who meet the ECMO indications should receive ECMO treatment as soon as possible if there is no contraindication.

ECMO mode selection: Venous‐venous ECMO (VV‐ECMO) is the most commonly used for respiratory support; venous‐arterial ECMO (VA‐ECMO) is used for patients who need both respiratory and circulatory support. Veno‐arterial‐venous EMCO (VAV‐ECMO) should be considered when differential hypoxia is developing in patients receiving VA‐ECMO. Lung protective ventilation strategies must be performed after ECMO treatment. Recommended initial settings: tidal volume <4–6 ml/kg ideal body weight, plateau pressure ≤25 cmH_2_O, driving pressure <15 cmH_2_O, PEEP 5–15 cmH_2_O, breathing rate 4–10 times/min, and FiO_2_ < 50%. For patients whose oxygenation is difficult to maintain, or those with a strong inspiratory effort, obvious consolidation of the gravity‐dependent areas of the lungs, or who needs active secretion drainage, prone position ventilation is recommended.

Children's cardiopulmonary compensatory ability is weaker than that of adults and more sensitive to hypoxia. They need to receive more active oxygen therapy and ventilation support strategies than adults. The indications should be appropriately relaxed. Routine recruitment of the lungs is not recommended.

#### 11.8.3 Circulation support

Critically ill patients can be complicated by shock. On the basis of adequate fluid resuscitation, vasoactive drugs should be used reasonably. Blood pressure, heart rate, and urine output changes, as well as lactic acid and base excess, should be closely monitored. Invasive hemodynamic monitoring should be used if necessary.

#### 11.8.4 Acute kidney injury (AKI) and renal replacement therapy

Critically ill patients can be complicated with AKI. Some factors that induced AKI such as drugs and low perfusion should be considered. Maintain water, electrolyte, and acid–base balance while treating the cause of AKI. Indications for continuous renal replacement therapy (CRRT) include (a) hyperkalemia, (b) severe acidosis, (c) pulmonary edema, or excessive fluid overload with ineffective diuretics.

#### 11.8.5 Children's MIS‐C

The treatment principle is multidisciplinary cooperation, anti‐inflammatory treatment, correcting shock and coagulation dysfunction, organ function support, and anti‐infection treatment when necessary. IVIG is the first choice for those without shock (2 g/kg). When the condition does not improve, add methylprednisolone 1–2 mg/kg/day or tozumab and other intensive treatment; IVIG combined with methylprednisolone 1–2 mg/kg/day is the first choice for patients with shock. Refractory severe children should be treated with high‐dose methylprednisolone shock (10–30 mg/kg/day) or combined with immunotherapy such as tozumab.

#### 11.8.6 Severe or critical cases with pregnancy

Multiple‐discipline consultation should be sought for risk assessment. The pregnancy should be terminated if necessary. The cesarean section is the first choice.

#### 11.8.7 Nutritional support

Nutritional risk assessment should be strengthened. Enteral nutrition with an energy of 25–30 kcal/kg/day and protein > 1.2 g/kg/day should be performed. Parenteral nutrition can be added if necessary. An intestinal microecological regulator can be used to maintain intestinal microecological balance and prevent secondary bacterial infection.

### 11.9 Traditional chinese medicine (TCM) therapy

This disease belongs to the plague in TCM, caused by epidemic pathogenic factors. According to the different local climate characteristics and individual states of illness and physical conditions, the following treatment protocol may vary. The use of overpharmacopoeia doses should be directed by a physician.

#### 11.9.1 During medical observation

*Clinical manifestation 1*: Fatigue and gastrointestinal discomfort

*Recommended Chinese patent medicine*: Huoxiang Zhengqi capsules (pills, liquid, or oral solution).

*Clinical manifestation 2*: Fatigue and fever

*Recommended Chinese patent medicines*: Jinhua Qinggan granules, Lianhua Qingwen capsules (granules), Shufeng Jiedu capsules (granules).

#### 11.9.2 During clinical treatment (confirmed cases)

##### 11.9.2.1 Qingfei Paidu decoction

*Scope of application*: It is suitable for mild, moderate, and severe patients, and can be used reasonably in combination with the actual situation of patients in the treatment of critically ill patients.

*Prescription composition*: Ma Huang (Ephedrae Herba) 9 g, Zhi Gan Cao (Glycyrrhizae Radix) 6 g, Xing Ren (Armeniacae Semen) 9 g, Sheng Shi Gao (Gypsum fibrosum) (decocted first) 15‐30 g, Gui Zhi (Cinnamomi Ramulus) 9 g, Ze Xie (Alismatis Rhizoma) 9 g, Zhu Ling (Polyporus) 9 g, Bai Zhu (Atractylodis macrocephalae Rhizoma) 9 g, Fu Ling (Poria) 15 g, Chai Hu (Bupleuri Radix) 16 g, Huang Qin (Scutellariae Radix) 6 g, Jiang Ban Xia (Pinellinae Rhizoma Praeparatum) 9 g, Sheng Jiang (Zingiberis Rhizoma recens) 9 g, Zi Wan (Asteris Radix) 9 g, Kuan Dong Hua (Farfarae Flos) 9 g, She Gan (Belamcandae Rhizoma) 9 g, Xi Xin (Asari Radix et Rhizoma) 6 g, Shan Yao (Dioscoreae Rhizoma) 12 g, Zhi Shi (Aurantii Fructus immaturus) 6 g, Chen Pi (Citri reticulatae Pericarpium) 6 g, Huo Xiang (Pogostemonis Herba) 9 g.

*Suggested use*: TCM decoction pieces for decocting in water. One dose daily with half of the dose taken in the morning, while the other half in the evening (40 min after meal) with warm water. Three days make a course of treatment.

If conditions permit, the patient can take half a bowl of rice soup each time after taking the medicine and can take up to one bowl if the patient has a dry tongue and is deficient in bodily fluids (Note: If the patient does not have a fever, the amount of gypsum should be little. If having a fever or high fever, the amount of gypsum can be increased). If the symptoms improve but do not fully recover, then take the second course of treatment. If the patient has special conditions or other underlying diseases, the prescription of the second course of treatment can be modified based on the actual situation and the medicine should be discontinued when the symptoms disappear.

*Suggested use of Qingfei Paidu granule*: TCM decoction pieces for decocting in boiled water. Two bags per day, twice daily, for 3–6 days.

##### 11.9.2.2 Mild cases

a. Cold‐dampness and stagnation lung syndrome

*Clinical manifestations*: Fever, fatigue, sore body, cough, expectoration, chest tightness, suffocation, loss of appetite, nausea, vomiting, sticky stools. The tongue has a thin fat tooth mark or is light red, the coating is white thick rot or white greasy, and the pulse is soggy or slippery.

*Recommended prescription*: Epidemic due to cold‐dampness formula.

*Prescription composition*: Sheng Ma Huang (Ephedrae Herba) 6 g, Sheng Shi Gao (Gypsum fibrosum) 15 g, Xing Ren (Armeniacae semen) 9 g, Qiang Huo (Notopterygii rhizoma seu Radix) 15 g, Ting Li Zi (Lepidii/Descurainiae Semen) 15 g, Guan Zhong (Cyrtomii rhizoma) 9 g, Di Long (Pheretima) 15 g, Xu Chang Qing (Cynanchi paniculati Radix) 15 g, Huo Xiang (Pogostemonis Herba) 15 g, Pei Lan (Eupatorii Herba) 9 g, Cang Zhu (Atractylodis Rhizoma) 15 g, Yun Ling (Poria) 45 g, Sheng Bai Zhu (*Atractylodis macrocephalae* Rhizoma) 30 g, Jiao San Xian (Jiao Shan Zha (Crataegi Fructus), Jiao Shen Qu (Massa medicate fermentata), and Jiao Mai Ya (Hordei Fructus germinatus) 9 g each, Hou Po (Magnoliae officinalis Cortex) 15 g, Jiao Bing Lang (*A. semen*) 9 g, Wei Cao Guo (Tsaoko Fructus) 9 g, Sheng Jiang (Zingiberis Rhizoma recens) 15 g.

*Suggested use*: One dose daily, boiled with 600 ml water, taking 1/3 of the dose in the morning, at noon, and in the evening, respectively, before a meal.

Epidemic due to cold‐dampness formula can be used on moderate patients.

b. Dampness and heat‐accumulation lung syndrome

*Clinical manifestations*: Low or no fever, slight chills, fatigue, heavy head and body, muscle soreness, dry cough, sore throat, dry mouth without desire of drinking much water, or accompanied by chest tightness, no sweat or sweating, or vomiting and loss of appetite, diarrhea or sticky stool. The tongue is reddish, the coating is white, thick, and greasy or thin yellow, and the pulse is slippery or soggy.

*Recommended prescription*: Bing Lang (*Arecae semen*) 10 g, Cao Guo (Tsaoko Fructus) 10 g, Hou Po (Magnoliae officinalis Cortex) 10 g, Zhi Mu (*Anemarrhenae rhizoma*) 10 g, Huang Qin (Scutellariae Radix) 10 g, Chai Hu (Bupleuri Radix) 10 g, Chi Shao (Paeoniae Radix Rubra) 10 g, Lian Qiao (Forsythiae Fructus) 15 g, Qing Hao (*Artemisiae annuae* Herba) (added later) 10 g, Cang Zhu (Atractylodis Rhizoma) 10 g, Da Qing Ye (Isatidis Folium) 10 g, Sheng Gan Cao (Glycyrrhizae Radix) 5 g.

*Suggested use*: One dose daily, boiled with 400 ml water, taking half of the dose in the morning and the other half in the evening.

*Recommended Chinese patent medicine*: Jinhua qinggan granule, Lianhua Qingwen capsule (granule). Jinhua Qinggan granule: take it with boiled water, one to two bags at a time, three times a day. The course of treatment is 5–7 days. Lianhua Qingwen granule: oral. One bag at a time, three times a day. The course of treatment is 7–10 days. Lianhua Qingwen capsule: oral. Four capsules at a time, three times a day.

*Recommended acupuncture points for acupuncture treatment*: Hegu, Houxi, yinlingquan, Taixi, Feishu, and Pishu. Acupuncture method: select three acupoints each time. Acupuncture adopts the method of flat tonic and flat catharsis. The degree of Qi is obtained. Keep the needle for 30 min once a day.

c. Dampness and heat‐accumulation lung syndrome

*Clinical manifestations*: low or no fever, slight chills, fatigue, heavy head and body, muscle soreness, dry cough, sore throat, dry mouth without desire of drinking much water, or accompanied by chest tightness, no sweat or sweating, or vomiting and loss of appetite, diarrhea or sticky stool. The tongue is reddish, the coating is white, thick, and greasy or thin yellow, and the pulse is slippery or soggy.

*Recommended prescription*: Bing Lang (*A. semen*) 10 g, Cao Guo (Tsaoko Fructus) 10 g, Hou Po (Magnoliae officinalis Cortex) 10 g, Zhi Mu (*A. rhizoma*) 10 g, Huang Qin (Scutellariae Radix) 10 g, Chai Hu (Bupleuri Radix) 10 g, Chi Shao (Paeoniae Radix rubra) 10 g, Lian Qiao (*Forsythiae Fructus*) 15 g, Qing Hao (*A. annuae* Herba) (added later) 10 g, Cang Zhu (Atractylodis Rhizoma) 10 g, Da Qjng Ye (Isatidis Folium) 10 g, Sheng Gan Cao (Glycyrrhizae Radix) 5 g.

*Suggested use*: One dose daily, boiled with 400 ml water, taking half of the dose in the morning and the other half in the evening.

*Recommended Chinese patent medicine*: Jinhua qinggan granule, Lianhua Qingwen capsule (granule). Jinhua Qinggan granule: take it with boiled water, one to two bags at a time, three times a day. The course of treatment is 5–7 days. Lianhua Qingwen granule: oral. One bag at a time, three times a day. The course of treatment is 7–10 days. Lianhua Qingwen capsule: oral. four capsules at a time, three times a day.

*Recommended acupuncture points for acupuncture treatment*: Hegu, Houxi, yinlingquan, Taixi, Feishu, and Pishu. Acupuncture method: select three acupoints each time. Acupuncture adopts the method of flat tonic and flat catharsis. The degree of Qi is obtained. Keep the needle for 30 min once a day.

##### 11.9.2.3 Moderate cases

a. Dampness and stagnation lung syndrome

*Clinical manifestations*: Fever, cough and scanty sputum, yellow sputum, suffocation, shortness of breath, bloating, and constipation. The tongue is dark red and fat, the coating is greasy or yellow, and the pulse is slippery or stringy.

*Recommended prescription*: Lung‐diffusing and toxin‐resolving formula

*Prescription composition*: Ma Huang (Ephedrae Herba) 6 g, Ku Xing Ren (Armeniacae Semen) 15 g, Sheng Shi Gao (Gypsum fibrosum) 30 g, Sheng Yi Yi Ren (Coicis Semen) 30 g, Mao Cang Zhu (Atractylodis Rhizoma) 10 g, Guang Huo Xiang (Pogostemonis Herba) 15 g, Qing Hao Cao (*A. annuae* Herba) 12 g, Hu Zhang (Polygoni cuspidati Rhizoma) 20 g, Ma Bian Cao (Verbenae Herba) 30 g, Gan Lu Gen (Phragmitis Rhizoma) 30 g, Ting Li Zi (Lepidii/Descurainiae Semen) 15 g, Hua Ju Hong (Citri grandis Exocarpium rubrum) 15 g, Gan Cao (Glycyrrhizae Radix) 10 g.

*Suggested use*: One dose daily, boiled with 400 ml water, taking half of the dose in the morning and the other half in the evening.

*Recommended Chinese patent medicine*: Xuanfei Baidu granule.

*Recommended use*: Take it with boiled water, one bag at a time, twice a day. The course of treatment is 7–14 days, or follow the doctor's advice.

b. Cold‐dampness lung syndrome

*Clinical manifestations*: Low fever, submerged fever or absence of fever, dry cough, scanty sputum, fatigue, chest tightness, stuffy and full sensation in the stomach, or nausea. The tongue is pale or red, the coating is white or greasy, and the pulse is soggy.

*Recommended prescription*: Cang Shu (Atractylodis Rhizoma) 15 g, Chen Pi (Citri reticulatae Pericarpium) 10 g, Hou Po (Magnoliae officinalis Cortex) 10 g, Huo Xiang (Pogostemonis Herba) 10 g, Cao Guo (Tsaoko Fructus) 6 g, Sheng Ma Huang (Ephedrae Herba) 6 g, Qiang Huo (Notopterygii Rhizoma seu Radix) 10 g, Sheng Jiang (Zingiberis Rhizoma recens) 10 g, Bing Lang (*A. semen*) 10 g.

*Suggested use*: One dose daily, boiled with 400 ml water, taking half of the dose in the morning and the other half in the evening.

c. Plague poison and dryness syndrome

*Clinical manifestations*: Cold aversion, fever, muscle soreness, runny nose, dry cough, sore throat, pharyngeal itching, dry mouth, dry throat, constipation, light red tongue, less fluid, thin white or dry coating, and tight pulse.

*Recommended prescription*: Lung‐diffusing, dryness moistening, and toxin‐resolving formula

*Prescription composition*: Ma Huang (Ephedrae Herba) 6 g, Xing Ren (Armeniacae Semen) 15 g, Chai Hu (Radix Bupleuri)12 g, Sha Seng (Radix Ginseng) 15 g, Mai Dong (*Ophiopogon japonicus*) 15 g, Xuan Seng (Radix Scrophulariae) 15 g, Bai Zhi (Radix Angelicae dahuricae) 10 g, Qiang Huo (notopterygium) 15 g, Sheng Ma (Cimicifuga) 8 g, Sang Ye (Mori Folium) 15 g, Huang Cen (Scutellaria baicalensis) 10 g, Sang Bai Pi (mulberry bark) 15 g, Sheng Shi Gao (Gypsum fibrosum) 20 g.

*Suggested use*: One dose daily, boiled with 400 ml water, taking half of the dose in the morning and the other half in the evening.

*Recommended Chinese patent medicine*: Jinhua Qinggan granule, Lianhua Qingwen capsule (granule). Jinhua Qinggan granule: oral. One to two bags at a time, three times a day. The course of treatment is 5–7 days. Lianhua Qingwen granule: oral. One bag at a time, three times a day. The course of treatment is 7–10 days. Lianhua Qingwen capsule: oral. Four capsules at a time, three times a day.

*Recommended acupuncture points for acupuncture treatment*: Neiguan, Kongzi, Quchi, Qihai, yinlingquan, and Zhongwan.

*Acupuncture method*: Select three acupoints each time. Acupuncture adopts the method of flat tonic and flat catharsis. The degree of Qi is obtained. Keep the needle for 30 min, once a day.

##### 11.9.2.4 Severe cases

a. Plague poison and lung‐closing syndrome

*Clinical manifestations*: Fever, flushing, cough, yellowish phlegm, or blood in sputum, wheezing, shortness of breath, tiredness, fatigue, dryness, bitterness, stickiness in the mouth, nausea, loss of appetite, poor stool, and short urination. The tongue is red, the coating is yellow greasy, and the pulse is slippery.

*Recommended prescription*: Dampness‐removing and toxin‐resolving formula

*Prescription composition*: Sheng Ma Huang (Ephedrae Herba) 6 g, Xing Ren (Armeniacae Semen) 9 g, Sheng Shi Gao (Gypsum fibrosum) 15 g, Gan Cao (Glycyrrhizae Radix) 3 g, Huo Xiang (Pogostemonis Herba) (added later) 10 g, Hou Po (Magnoliae officinalis Cortex) 10 g, Cang Zhu (Atractylodis Rhizoma) 15 g, Cao Guo (Tsaoko Fructus) 10 g, Fa Ban Xia (Pinellinae Rhizoma Praeparatum) 9 g, Fu Ling (Poria) 15 g, Sheng Da Huang (Rhei Radix et Rhizoma) (added later) 5 g, Sheng Huang Qi (Astragali Radix) 10 g, Ting Li Zi (Lepidii/Descurainiae Semen) 10 g, Chi Shao (Paeoniae Radix rubra) 10 g.

*Suggested use*: One or two doses daily, boiled with 100–200 ml water, finish the dose(s) two to four times across the day, oral or nasal feeding.

*Recommended Chinese patent medicine*: Huashi Baidu granule.

*Huashi Baidu granule*: take it with boiled water, two bags at a time, two times a day, or follow the doctor's advice.

b. Blazing of both qi and ying syndrome

*Clinical manifestations*: Hot fever, thirst, shortness of breath, delirium and unconsciousness, blurred vision, or spotted rash, or hematemesis, epistaxis, or convulsions in the limbs. The tongue is crimson with little or no coating. The pulse is deep, fine and rapid, or floating, large and rapid.

*Recommended prescription*: Sheng Shi Gao (Gypsum Fibrosum) (decocted first) 30–60 g, Zhi Mu (*A. rhizoma*) 30 g, Sheng Di (Rehmanniae Radix) 30–60 g, Shui Niu Jiao (Bubali Cornu) (decocted first) 30 g, Chi Shao (Paeoniae Radix rubra) 30 g, Xuan Shen (Scrophulariae Radix) 30 g, Lian Qiao (*Forsythiae Fructus*) 15 g, Dan Pi (Moutan Cortex) 15 g, Huang Lian (Coptidis Rhizoma) 6 g, Zhu Ye (Phyllostachys nigrae Folium) 12 g, Ting Li Zi (Lepidii/Descurainiae Semen) 15 g, Sheng Gan Cao (Glycyrrhizae Radix) 6 g.

*Suggested use*: One dose per day, decoction, first decoct Sheng Gan Cao (Glycyrrhizae Radix) and Shui Niu Jiao (Bubali Cornu), then apply other pieces, boiled with 100–200 ml water, finish the dose(s) in two to four times across the day, orally or nasally.

*Recommended Chinese patent medicines*: Xiyanping injection, Xuebijing injection, Reduning injection, Tanreqing injection, Xingnaojing injection. Drugs with similar efficacy can be selected according to individual conditions or can be used in combination according to clinical symptoms. TCM injection can be used in combination with TCM decoction.

*Recommended acupuncture points for acupuncture treatment*: Dazhui, Feishu, Pishu, Taixi, Lieke, and Taichong. Acupuncture method: apply on Beishu and limb acupoints, acupuncture for tonifying and reducing diarrhea, keep the needle for 30 min each time, once in a day.

##### 11.9.2.5 Critically ill cases

Internal blockage and external desertion syndrome

*Clinical manifestations*: Dyspnea, asthma, or mechanical ventilation needed, fainting, irritability, sweating, cold limbs, dark purple tongue, thick greasy or dry coating, and large floating pulse without root.

*Recommended prescription*: Ren Shen (Ginseng Radix) 15 g, Hei Shun Pian (Aconiti Radix lateralis praeparata) (decocted first) 10 g, Shan Zhu Yu (Corni Fructus) 15 g, delivered with Suhexiang Pill or Angong Niuhuang Pill.

*For patients on mechanical ventilation with abdominal distention or constipation*: 5–10 g of Sheng Da Huang (Rhei Radix et Rhizoma). For patients with human‐machine asynchronization: 5–10 g of Sheng Da Huang (Rhei Radix et Rhizoma) and 5–10 g of Mang Xiao (Natrii Sulfas) while administering sedatives and muscle relaxants.

*Recommended Chinese patent medicines*: Xuebijing injection, reduning injection, Tanreqing injection, Xingnaojing injection, Shenfu injection, Shengmai injection, Shenmai injection. Drugs with similar efficacy can be selected according to individual conditions or can be used in combination according to clinical symptoms. TCM injection can be used in combination with TCM decoction.

*Note*: Recommended usage of TCM injections for severe and critical cases

The use of TCM injections follows the principle of starting from a small dose and gradually adjusting the dosage according to the instructions of the drug. The recommended usage is as follows:

*Viral infection or combined mild bacterial infection*: 0.9% sodium chloride injection 250 ml plus Xiyanping injection 100 mg bid, or 0.9% sodium chloride injection 250 ml Reduning injection 20 ml, or 0.9% sodium chloride injection 250 ml plus Tanreqing injection 40 ml bid.

*High fever with disturbance of consciousness*: 250 ml of 0.9% sodium chloride injection and 20 ml bid of Xingnaojing injection.

*Systemic inflammatory response syndrome or/and multiple organ failure*: 250 ml of 0.9% sodium chloride injection and 100 ml of Xuebijing injection bid.

*Immunosuppression*: 250 ml of glucose injection with 100 ml of Shenmai injection or 20–60 ml of Shengmai injection, bid.

*Recommended acupuncture points for acupuncture treatment*: Taixi, Tanzhong, Guanyuan, Baihui, Zusanli. Acupuncture method: select the above acupoints, acupuncture for tonifying and reducing diarrhea, keep the needle for 30 min, once a day.

##### 11.9.2.6 Convalescent period

a. Lung and spleen qi deficiency syndrome

*Clinical manifestations*: Shortness of breath, fatigue, anorexia, nausea, fullness, loose stool, and uneasiness. The tongue is pale and greasy.

*Recommended prescription*: Fa Ban Xia (Pinellinae Rhizoma Praeparatum) 9 g, Chen Pi (Citri reticulatae Pericarpium) 10 g, Dang Shen (Codonopsis Radix) 15 g, Zhi Huang Qi (Astragali Radix) 30 g, Chao Bai Zhu (Atractylodis macrocephalae Rhizoma) 10 g, Fu Ling (Poria) 15 g, Huo Xiang (Pogostemonis Herba) 10 g, Sha Ren (AmomiFructus) (added later) 6 g, Gan Cao (Glycyrrhizae Radix) 6 g.

*Suggested use*: One dose per day, boiled with 400 ml of water, taking half of the dose in the morning and the other half in the evening.

b. Deficiency of both qi and yin syndrome.

*Clinical manifestations*: fatigue, shortness of breath, dry mouth, thirst, palpitations, sweating, poor appetite, low or no fever, dry cough, dry tongue, fine or weak pulse.

*Recommended prescription*: Nan Sha Shen (Adenophorae Radix) 10 g, Bei Sha Shen (Glehniae Radix) 10 g, Mai Dong (Ophiopogonis Radix) 15 g, Xi Yang Shen (Panacis quinquefolii Radix) 6 g, Wu Wei Zi (Schisandrae Fructus) 6 g, Sheng Shi Gao (Gypsum fibrosum) 15 g, Dan Zhu Ye (Lophatheri Herba) 10 g, Sang Ye (Mori Folium) 10 g, Lu Gen (Phragmitis Rhizoma) 15 g, Dan Shen (Salviae miltiorrhizae Radix) 15 g, Sheng Gan Cao (Glycyrrhizae Radix) 6 g.

*Suggested use*: One dose per day, boiled with 400 ml of water, taking half of the dose in the morning and the other half in the evening.

*Recommended acupuncture points for acupuncture treatment*: Zusanli (moxibustion), Baihui, and Taixi. Acupuncture method: select the above acupoints, acupuncture for tonifying and reducing diarrhea, keep the needle for 30 min, once a day. Point selection of separated moxibustion: Dazhui, Feishu, Pishu, Kongzi, 40 min each time, once a day.

#### 11.9.3 TCM treatment for children

The TCM syndrome characteristics and core pathogenesis of children are basically the same as those of adults. The treatment is based on the adult TCM treatment scheme, with consideration of the clinical symptoms and the physiological characteristics of children. Children can choose to use Chinese patent medicine according to syndrome differentiation.

### 11.10 Early rehabilitation therapy

Attention should be given to early rehabilitation of patients with COVID‐19, and early rehabilitation training and intervention for respiratory and physical function, and psychological disorders should be actively implemented to restore physical fitness and immunity as much as possible.

## 12 NURSING

According to the patient's condition, nurses must clarify the key points of care and maintain proper basic care. In critically ill patients, close observation of the patient's vital signs, state of consciousness, and monitoring of blood oxygen saturation must be implemented. Critically ill patients must have 24‐h continuous ECG monitoring, measurements of patient's heart rate, RR, blood pressure, and SpO_2_ every hour, as well as measuring and recording body temperature every 4 h. Venous access must be done correctly, and all conduits must be unobstructed and properly fixed. Bedridden patients must change their positions regularly to prevent pressure sores. Implementation of noninvasive mechanical ventilation, invasive mechanical ventilation, artificial airway, prone position ventilation, sedation and analgesia, and ECMO is done in accordance with nursing regulations. Special attention is needed for patients' oral care and fluid inflow and outflow management, as well as aspiration prevention in patients with invasive mechanical ventilation. Psychological assessment should be done for conscious patients and proper care provided.

## 13 DISCHARGE CRITERIA AND PRECAUTIONS AFTER DISCHARGE

### 13.1 Criteria for isolation lift

The isolation can be lifted, if (a) the *C*_t_ value of the N gene and ORF gene of two consecutive 2019‐nCoV nucleic acid tests of the mild case are above 35 (Quantitative Fluorescence PCR with a Cycle threshold value of 40, at least 24 h apart) or (b) results of two consecutive 2019‐nCoV nucleic acid tests are negative (Quantitative Fluorescence PCR method with a Cycle threshold value of below 35, at least 24 h apart).

### 13.2 Discharge criteria

a. Body temperature returns to normal for more than 3 days;
b. Significantly improved respiratory symptoms;
c. Lung imaging shows significant improvement in acute exudative lesions;
d. The *C*_t_ value of the N gene and ORF gene of two consecutive 2019‐nCoV nucleic acid tests are above 35 (quantitative fluorescence PCR with a cycle threshold value of 40, at least 24 h apart), or the results of two consecutive 2019‐nCoV nucleic acid tests are negative (quantitative fluorescence PCR method with a cycle threshold value of below 35, at least 24 h apart).

Those who meet the above conditions can be discharged.

### 13.3 Precautions after discharge

It is recommended to continue 7 days of health monitoring at home after discharge from the hospital, wear masks, live in a single room with good ventilation, reduce close contact with family members, eat and drink separately, pay attention to hand hygiene, and avoid going out for public activities.

## 14 PATIENT TRANSFER

Healthcare providers are required to follow the *Work Plan for the Transfer of Pneumonia Cases Infected by the 2019‐nCoV (Trial Version 2)* issued by the National Health Commission.

## 15 CONTROL OF NOSOCOMIAL INFECTION IN MEDICAL INSTITUTIONS

Healthcare facilities shall strictly follow the requirements of the *Technical Guideline for the Prevention and Control of 2019‐nCoV Infection in Health Care Settings (Third Edition)* issued by the National Health Commission.

## 16 DISEASE PREVENTION

### 16.1 COVID‐19 vaccines

COVID‐19 vaccines can reduce the risk of infection, and prevent severe disease and death. Those eligible ones for vaccination should all have the vaccine. Those eligible ones for the boost COVID‐19 vaccine should take the boost dose in time.

### 16.2 General measures

People are encouraged to maintain good personal and environmental hygiene, balanced nutrition, moderate exercise, adequate rest, and avoid excessive fatigue. Health literacy should be improved, hygienic habits developed, and social distancing maintained. People should wash hands frequently, wear masks, cover their mouth and nose when sneezing or coughing, and use serving utensils when two or more are dining together. Indoor spaces should be well ventilated. Everyone should protect themselves through appropriate measures and must visit a fever clinic if respiratory symptoms occur. Those who have recently traveled to high‐risk areas or have an exposure history with confirmed or suspected cases should have the nucleic acid test done.

The official notice of the release and the official Diagnosis and Treatment Protocol for COVID‐19 Patients (Trial Version 9) can be found at:

[News of the Release, Letter No. 71 [2022] of the National Health Commission] http://www.gov.cn/zhengce/zhengceku/2022-03/15/content_5679257.htm

[The Official Protocol (Trial Version 9)] https://www.gov.cn/zhengce/zhengceku/2022-03/15/5679257/files/49854a49c7004f4ea9e622f3f2c568d8.pdf

## AUTHOR CONTRIBUTION

Group author ‐ equal contribution.

## ACKNOWLEDGEMENT

Not applicable.

## CONFLICT OF INTEREST

The author declares no conflict of interest.

## DATA AVAILABILITY STATEMENT

No data involved.

## ETHICS STATEMENT

Not applicable.

## INFORMED CONSENT

Not applicable.

